# Neuroinflammation and J2 prostaglandins: linking impairment of the ubiquitin-proteasome pathway and mitochondria to neurodegeneration

**DOI:** 10.3389/fnmol.2014.00104

**Published:** 2015-01-13

**Authors:** Maria E. Figueiredo-Pereira, Patricia Rockwell, Thomas Schmidt-Glenewinkel, Peter Serrano

**Affiliations:** ^1^Department of Biological Sciences, Hunter College, The Graduate School and University Center, City University of New YorkNew York, NY, USA; ^2^Department of Psychology, Hunter College, The Graduate School and University Center, City University of New YorkNew York, NY, USA

**Keywords:** J2 prostaglandins, neuroinflammation, UPP, mitochondria, neurodegeneration

## Abstract

The immune response of the CNS is a defense mechanism activated upon injury to initiate repair mechanisms while chronic over-activation of the CNS immune system (termed neuroinflammation) may exacerbate injury. The latter is implicated in a variety of neurological and neurodegenerative disorders such as Alzheimer and Parkinson diseases, amyotrophic lateral sclerosis, multiple sclerosis, traumatic brain injury, HIV dementia, and prion diseases. Cyclooxygenases (COX-1 and COX-2), which are key enzymes in the conversion of arachidonic acid into bioactive prostanoids, play a central role in the inflammatory cascade. J2 prostaglandins are endogenous toxic products of cyclooxygenases, and because their levels are significantly increased upon brain injury, they are actively involved in neuronal dysfunction induced by pro-inflammatory stimuli. In this review, we highlight the mechanisms by which J2 prostaglandins (1) exert their actions, (2) potentially contribute to the transition from acute to chronic inflammation and to the spreading of neuropathology, (3) disturb the ubiquitin-proteasome pathway and mitochondrial function, and (4) contribute to neurodegenerative disorders such as Alzheimer and Parkinson diseases, and amyotrophic lateral sclerosis, as well as stroke, traumatic brain injury (TBI), and demyelination in Krabbe disease. We conclude by discussing the therapeutic potential of targeting the J2 prostaglandin pathway to prevent/delay neurodegeneration associated with neuroinflammation. In this context, we suggest a shift from the traditional view that cyclooxygenases are the most appropriate targets to treat neuroinflammation, to the notion that J2 prostaglandin pathways and other neurotoxic prostaglandins downstream from cyclooxygenases, would offer significant benefits as more effective therapeutic targets to treat chronic neurodegenerative diseases, while minimizing adverse side effects.

## Introduction

Chronic neuroinflammation is recognized as a primary mechanism involved in the pathogenesis of a variety of neurodegenerative disorders including Alzheimer, Parkinson, and Huntington diseases as well as amyotrophic lateral sclerosis (Wyss-Coray and Mucke, 2002; Liu and Hong, 2003; Glass et al., 2010; Herrup, 2010). Neuroinflammation is an active process detectable in the earliest stages of these diseases (Zagol-Ikapitte et al., 2005; Liang et al., 2007; Yoshiyama et al., 2007). The neurotoxicity associated with inflammation makes it a potential risk factor in their pathogenesis. Characterizing the self-perpetuating cycle of inflammatory processes involving microglia and astrocytes in the brain that drives the slow progression of neurodegeneration could be critical for preventing/arresting these devastating disorders (Schwab and McGeer, 2008; Herrup, 2010).

**Figure 1:**
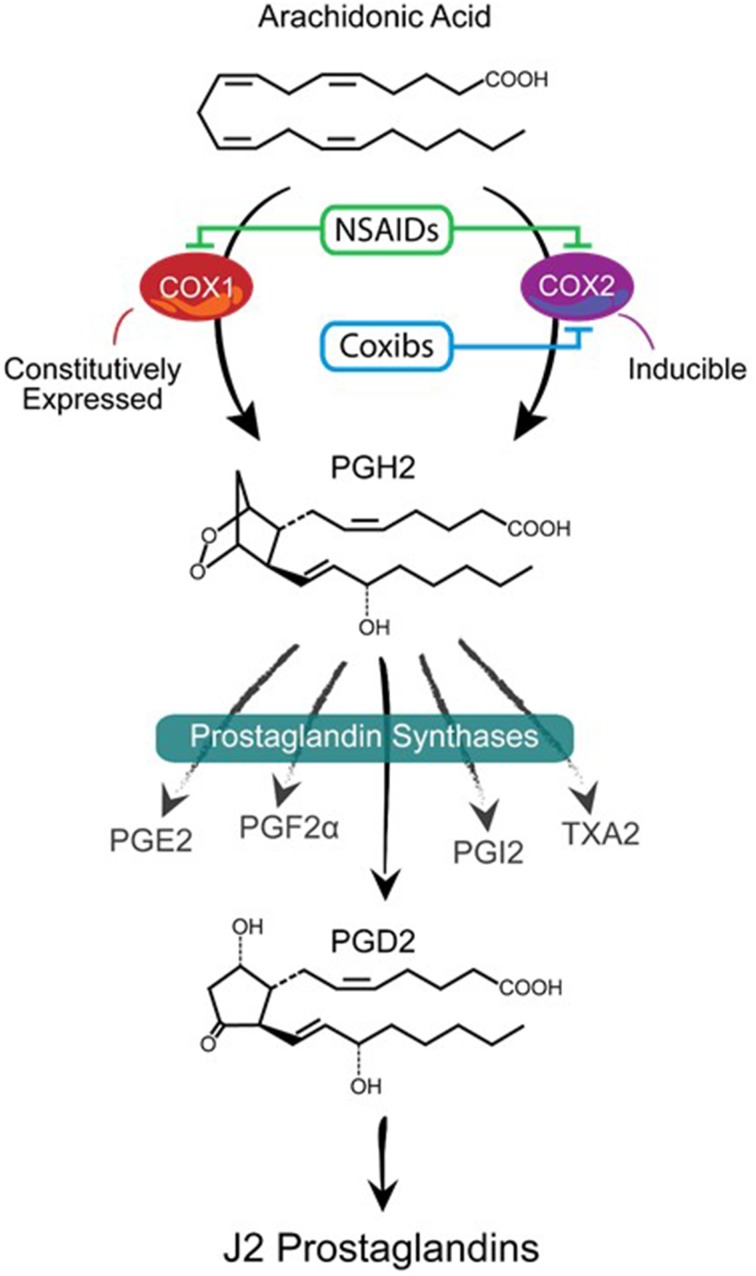
**Prostanoid biosynthetic pathway**. Arachidonic acid is converted via a two-step process (cyclooxygenation and hydroperoxidation) by cyclooxygenase enzymes COX-1 or COX-2 into the unstable prostaglandin PGH2. COX-1 is constitutively expressed while COX-2 is mostly an inducible enzyme that is upregulated under stress conditions. Non-steroidal anti-inflammatory drugs (NSAIDs) block the activities of both enzymes while Coxibs are selective COX-2 inhibitors. PGH2 is then converted to prostanoid products (PGE2, PGF2α, PGD2, PGI2, and TXA2) by specific prostaglandin synthases that differ in their cell type distribution. Of these products, PGD2 is highly unstable (estimated brain half-life of 1.1 min) resulting in the non-enzymatic formation of J2 prostaglandins.

Major players in inflammation are the cyclooxygenases COX-1 and COX-2 (Figure 1), which function as homodimers and are key enzymes in the biosynthesis of prostaglandins (Smyth et al., 2009). Although the brain expresses COX-1 and COX-2 under normal physiological conditions, it is clear that cyclooxygenases are implicated in neurodegeneration (Liang et al., 2007; Bartels and Leenders, 2010). COX-1 is generally viewed as being the homeostatic isoform, but studies suggest that it is actively involved in some forms of brain injury (Choi et al., 2009; Aid and Bosetti, 2011). The expression and activity of COX-2 are largely responsive to adverse stimuli, such as inflammation and physiologic imbalances (Yamagata et al., 1993). COX-2 activity is markedly induced in a range of neurodegenerative disorders subsequently leading to neuronal injury (Feng et al., 2003; Klivenyi et al., 2003; Teismann et al., 2003). COX-2 up-regulation following CNS injury is not restricted to neurons since COX-2 induction is also apparent in glia (Consilvio et al., 2004). Although many studies support the notion that COX-2 is involved in neurodegeneration, its contribution to the neurodegenerative process remains poorly defined. Inhibiting cyclooxygenases with non-steroidal anti-inflammatory drugs (NSAIDs) is being explored as a therapeutic strategy to mitigate chronic inflammation and prevent the onset/progression of neuropathology (Klegeris et al., 2007; Vlad et al., 2008). However, the effectiveness of NSAIDs could be counterproductive by blocking the generation of all prostaglandin products of cyclooxygenases (Figure 1).

Current animal and cell models of neurodegenerative diseases fail to address how prostaglandins redirect cellular events to promote neurodegeneration. This is a crucial gap since some prostaglandins are neuroprotective and others neurotoxic (Lucin and Wyss-Coray, 2009; Iadecola and Gorelick, 2005). Since prostaglandins act as potent local regulators of physiologic and pathologic pathways linked to CNS inflammation, elucidating the prostaglandin-dependent pathologic pathways will have a major impact on blocking neurotoxicity linked to chronic neuroinflammation with fewer undesirable side effects, and could lead to preventing/delaying neurodegeneration.

## Formation of J2 prostaglandins

**Figure 2:**
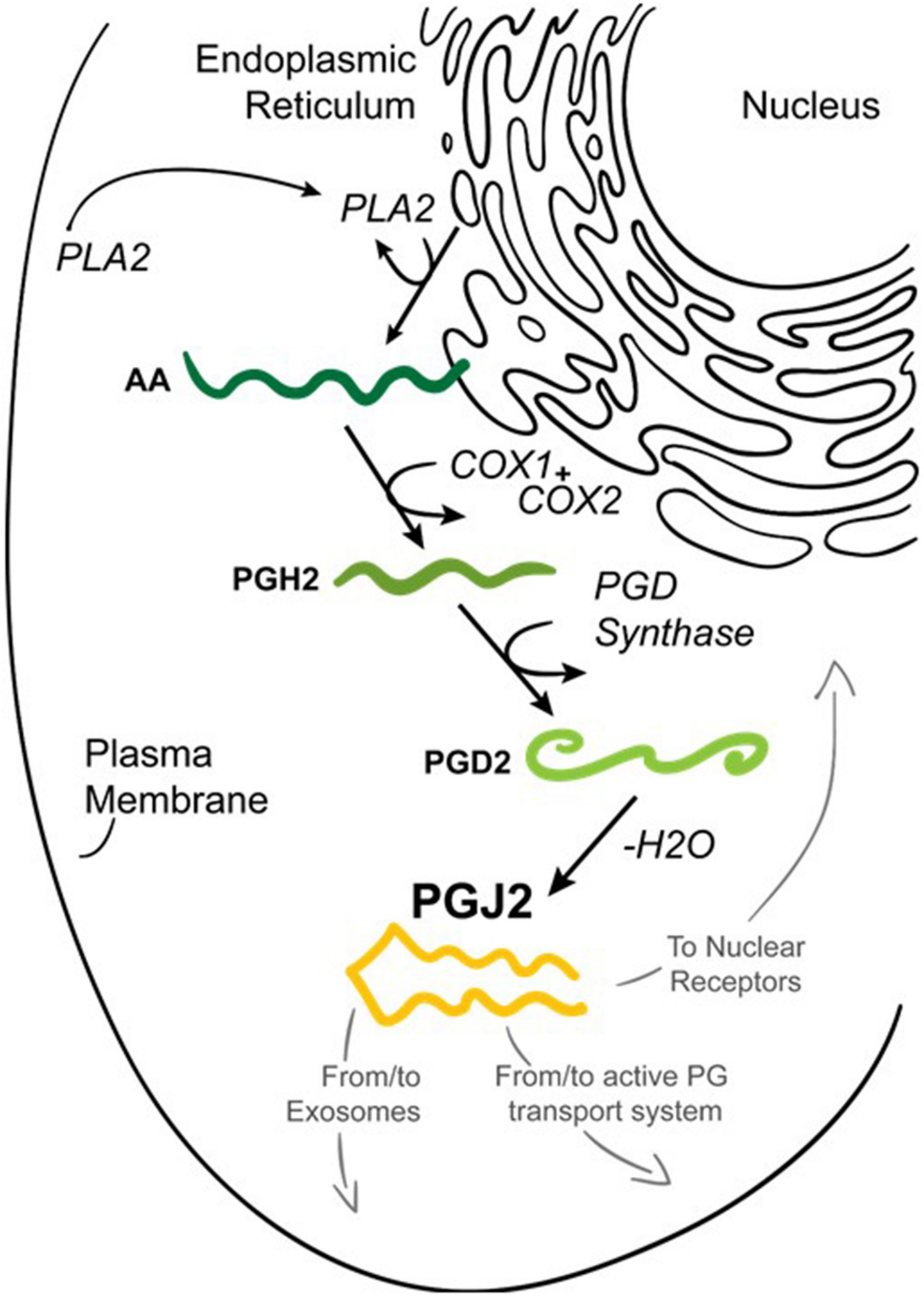
**Formation of prostaglandin J2 (PGJ2)**. Upon cell activation by mechanical trauma, cytokines, growth factors or other stressful stimuli, phospholipase A2 (PLA2) is recruited from the cytoplasm to intracellular membranes (nucleus or endoplasmic reticulum) to catalyze the hydrolysis of membrane sn-2 glycerophospholipids releasing arachidonic acid (AA, dark green). AA is converted by COX-1 or COX-2 to PGH2 (medium green) which is then converted to PGD2 (light green) by PGD synthase. PGD2 undergoes a non-enzymatic dehydration (–H_2_O) to biologically active PGJ2 (yellow). PGJ2 can be localized to exosomes, to transport systems or to nuclear receptors to mediate its function.

Prostaglandins (PGs) are a family of 20-carbon unsaturated fatty acids produced via the cyclooxygenase pathway in response to numerous extrinsic and intrinsic stimuli (Figure 2). The initial step in prostaglandin synthesis involves the hydrolysis of membrane sn-2 glycerophospholipids (phosphatidylcholine, phosphatidylethanolamine, and phosphatidylinositol) by phospholipase A2 (PLA2 group IVA) to release arachidonic acid (Tassoni et al., 2008; Smyth et al., 2009; Astudillo et al., 2012). PLA2 is activated by increased calcium levels and phosphorylation. This event leads to the translocation of PLA2 from the cytoplasm to intracellular membranes including the endoplasmic reticulum and nuclear envelope, to allow its access to arachidonic acid-containing phospholipid substrates (Shimizu et al., 2008).

Cyclooxygenases, which are bifunctional enzymes inserted into the ER and nuclear membranes, will then catalyze the cyclooxygenation of arachidonic acid to PGG2 followed by hydroperoxidation of PGG2 to PGH2 (Kulkarni et al., 2000; Smith et al., 2000; Simmons et al., 2004). PGH2 diffuses from the ER lumen through its membrane to the cytoplasm to be converted to more polar prostanoids via synthases localized on the cytoplasmic face of the ER (Schuster, 2002). The coupling of PGH2 synthesis with the respective downstream synthase enzymes that produce the different types of prostaglandins is intricately orchestrated in a tissue and/or cell specific manner (Funk, 2001).

**Figure 3:**
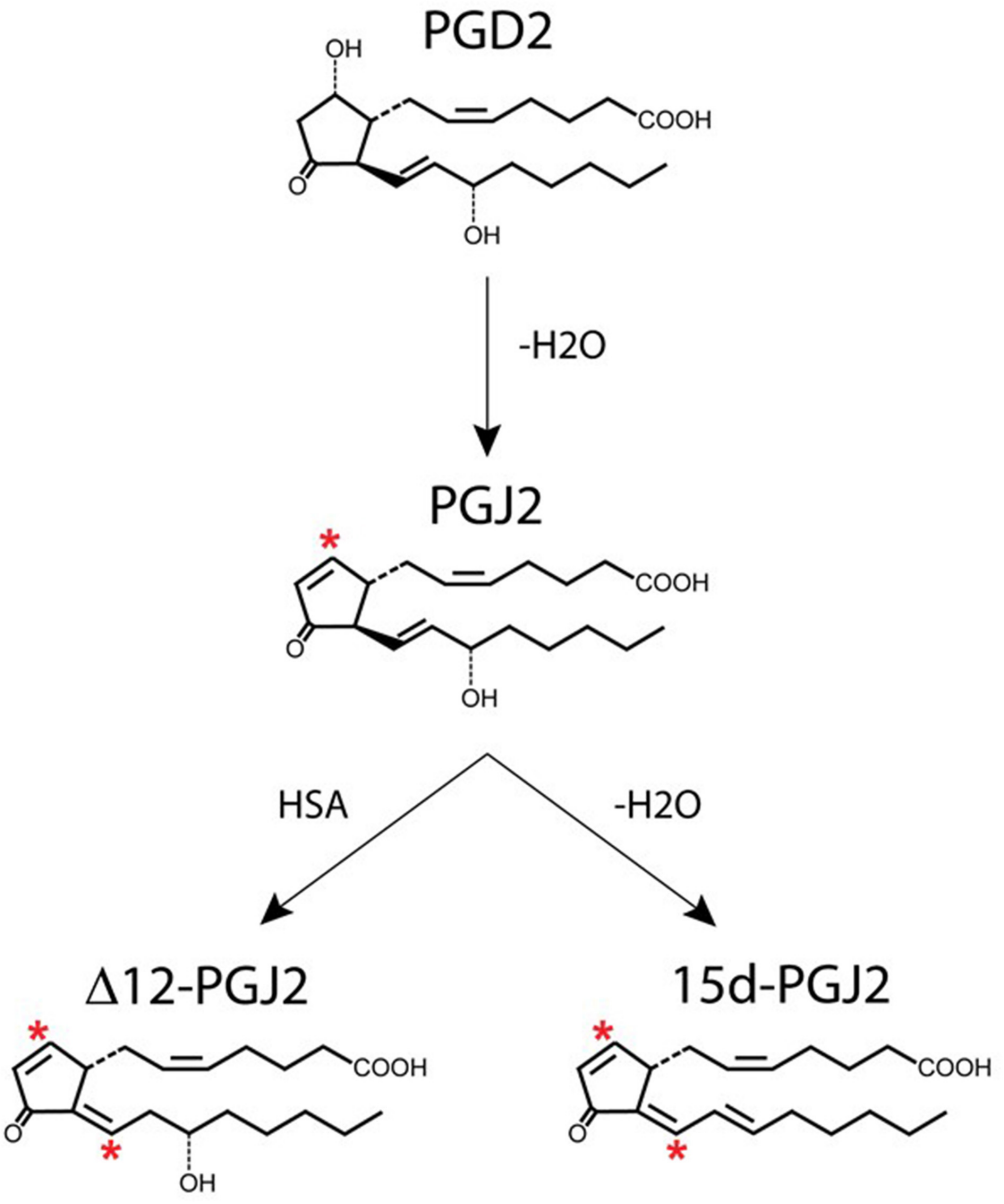
**Generation of J2 prostaglandins**. PGJ2 is generated by non-enzymatic dehydration of PGD2. The J2 metabolites Δ12-PGJ2 and 15d-PGJ2 are formed from PGJ2 either by reactions catalyzed by human serum albumin (HSA) or by dehydration (–H_2_O), respectively. Asterisks indicate α,β-unsaturated carbonyl groups.

J2 prostaglandins (Figure 3) are derived from PGD2, which is the most abundant prostaglandin in the brain (Ogorochi et al., 1984; Hertting and Seregi, 1989; Uchida and Shibata, 2008; Ricciotti and FitzGerald, 2011), and the one that changes the most under pathological conditions (Liang et al., 2005). PGD2 is produced by two distinct prostaglandin D2 synthases (PGDS), which carry out the isomerization of PGH2 to PGD2: (i) the hematopoietic enzyme (H-PGDS) and (ii) the lipocalin enzyme (L-PGDS) (Urade and Hayaishi, 2000; Urade and Eguchi, 2002). H-PGDS is a cytosolic protein found abundantly in mast cells, antigen presenting cells, and T helper type 2 (Th2) cells (Kanaoka and Urade, 2003). L-PGDS is localized in the CNS, heart and male genital organs (Urade and Eguchi, 2002). L-PGDS is one of the most abundant CSF proteins produced in the brain (Kanekiyo et al., 2007), representing 3% of total CSF protein (Xu and Venge, 2000). Secreted L-PGDS in the CSF has a dual function: it increases CSF-PGD2 levels (Scher and Pillinger, 2005) and also acts as a lipophilic-ligand carrier (Urade and Hayaishi, 2000), being a major endogenous Aβ-chaperone in the brain (Kanekiyo et al., 2007).

PGD2 is unstable and readily undergoes *in vivo* and *in vitro* non-enzymatic dehydration to generate the biologically active cyclopentenone J2 prostaglandins (Figure 3), which include PGJ2, Δ12-PGJ2, and 15-deoxy-Δ12,14-PGJ2 (15d-PGJ2) (Shibata et al., 2002; Uchida and Shibata, 2008; Gilroy, 2010). The half-life of PGD2 in the brain was estimated to be 1.1 min and in the blood 0.9 min (Suzuki et al., 1986).

PGJ2 and its metabolites are not stored in tissues or cells and their production increases with diverse stimuli. Prostaglandins are largely produced in the brain by activated microglia, reactive astrocytes and neurons. During CNS inflammation, these cells make large quantities of prostaglandins such as PGE2 and PGD2 (Liu et al., 2003) as well as J2 prostaglandins (Bernardo et al., 2003). For example, LPS-activated microglia in culture, produced ~3 ng/ml media of 15d-PGJ2 upon 72 h, and ~2 ng/ml of PGD2 upon 24 h (Bernardo et al., 2003). J2 prostaglandins have been detected *in vivo* in human body fluids (Hirata et al., 1988), human atherosclerotic plaques (Shibata et al., 2002) and tissues of patients with sporadic ALS (Kondo et al., 2002; Zhang et al., 2010). In addition, a range of studies showed that J2 prostaglandins are generated *in vivo* upon various conditions related to brain injury (see below).

## *In vivo* levels of J2 prostaglandins in the CNS

Prostaglandins are present in body fluids in the pico to nanomolar range reaching low micromolar levels at local sites of acute inflammation (Offenbacher et al., 1986; Hertting and Seregi, 1989). For example, in human airways PGD2 rose in 9 min to an average of 150-fold in five patients in response to an allergen (Murray et al., 1986). Moreover, exosomes, which are extracellular bioactive vesicles released from multivesicular bodies that mediate intercellular signaling (Subra et al., 2010), were found to contain a large panel of free fatty acids, including arachidonic acid and its derivatives, such as PGE2 and PGJ2 (Subra et al., 2010). In fact, the levels of these prostaglandins within exosomes was determined to be in the micromolar range, thus at concentrations capable of triggering prostaglandin-dependent biological effects (Subra et al., 2010).

J2 prostaglandins (Table 1) are bioactive cyclopentenone prostaglandins produced *in vivo* during inflammation (Rajakariar et al., 2007). Like their precursor, J2 prostaglandins can be considered some of the most abundant prostaglandins in the brain (Katura et al., 2010). For example, plasma levels of 15d-PGJ2 increased 12-fold and 23-fold in patients following acute stroke or with vascular risk factors and atherothrombotic infarcts, respectively (Blanco et al., 2005). In rodents, stroke (cerebral ischemia) and traumatic brain injury (TBI) elevate PGJ2 levels in the brain to concentrations similar to those shown to be neurotoxic *in vitro* (Kunz et al., 2002; Hickey et al., 2007; Liu et al., 2013a,b,c; Shaik et al., 2014). Accordingly, the *in vivo* concentration of free PGJ2 in the brain upon stroke and TBI, increases from almost undetectable to the 100 nM range (Liu et al., 2011, 2013a). These levels represent average brain concentrations, but it is predicted that local cellular and intracellular concentrations of J2 prostaglandins are much higher (Liu et al., 2013b). It is also clear that this is an underestimation of the overall J2 prostaglandin levels *in vivo*, as they bind covalently to proteins (see below), and therefore reported levels of free J2 prostaglandins do not represent their total amounts.

**Table 1:** ***In vivo* levels of J2 prostaglandins**.

**Pathology**	**Strain**	**Treatment**	**Region and (method)**	**Prostaglandin**	**Basal levels**	**Pathological levels**	**References**
**TBI**	Male Sprague-Dawley rats	Fluid percussion and controlled cortical contusion injuries	Cerebral cortex and hippocampus (LC-MS)	PGD2 (most abundant) and its metabolites detected	N/A	Increase (no quantification)	Kunz et al., 2002
**TBI**	Sprague-Dawley rats	Controlled cortical impact	Whole brain homogenates (ELISA)	PGE2	~12.5 ng/g	Four-fold increase to 50 ng/g	Hickey et al., 2007
**ALS**	Patients	N/A	Spinal motor neurons (immuno-histochemistry)	15d-PGJ2	N/A	N/A	Kondo et al., 2002; Zhang et al., 2010
**Stroke, vascular risk factors and atherothrombotic infarcts**	Patients (24 h after acute stroke)	N/A	Plasma (ELISA)	15d-PGJ2	~5.0 pg/ml	~60.5 pg/ml	Blanco et al., 2005
**Acute inflammation: peritonitus**	H-PGDS KO mice	Type A zymosan injection	Peritoneal cavity fluid (LC-MS/MS)	15d-PGJ2	500 pg/ml	Average 0.5–5 ng/ml	Rajakariar et al., 2007
**Arachidonic acid peroxidation**	Male Sprague-Dawley rats	Carbon tetrachloride (CCl4)	Liver (LC-MS/MS)	Deoxy J2 isoprostanes: 15d-PGJ2-like compounds	0.55 ± 0.21 ng/ g	6.4 ± 1.1 ng/g	Hardy et al., 2011
**Seizures**	Male Wistar rats	Kainic acid	Hippocampal tissue (immuno-histochemistry)	PGF2a	OD = 4.4	OD = 41.5	Takei et al., 2012
**Temporary focal ischemia**	Male Sprague-Dawley rats	Middle cerebral artery occlusion	Whole brain homogenates (quadrupole MS/MS)	15d-PGJ2	Sham: 0	23.72 nM	Liu et al., 2011
**Temporary focal ischemia**	Male Sprague-Dawley rats	Middle cerebral artery occlusion	Infarct penumbral brain regions (UPLC-MS/MS)	PGD2, PGJ2, A12-PGJ2, 15d-PGJ2	(nM) D2: 102.3 J2: 3.7 A12-J2: 0.7 15d-J2: 0	(nM) D2: 465.5 J2: 94.7 A12-J2: 18.6 15d-J2: 155.9	Liu et al., 2013a
**Global brain ischemia**	Male Sprague-Dawley rats	Asphyxial cardiac arrest	Hippocampus (UPLC-MS/MS)	PGE2, PGD2, PGJ2, 15d-PGJ2	(pmol/g) E2: ~100 D2: ~500 J2: ~40 15d-J2: 0	(pmol/g) E2: ~550 D2: ~3000 J2: ~160 15d-J2: ~16	Liu et al., 2013b
**Global brain ischemia**	Male Sprague-Dawley rats	Asphyxial cardiac arrest	Cerebral cortical tissue (UPLC-MS/MS)	PGE2, PGD2, PGJ2, 15d-PGJ2	N/A	(pmol/g) E2: 35.5 D2: 937.0 J2: 36.9 15d-J2: 18.4	Shaik et al., 2014

## Modes of action of J2 prostaglandins

**Figure 4:**
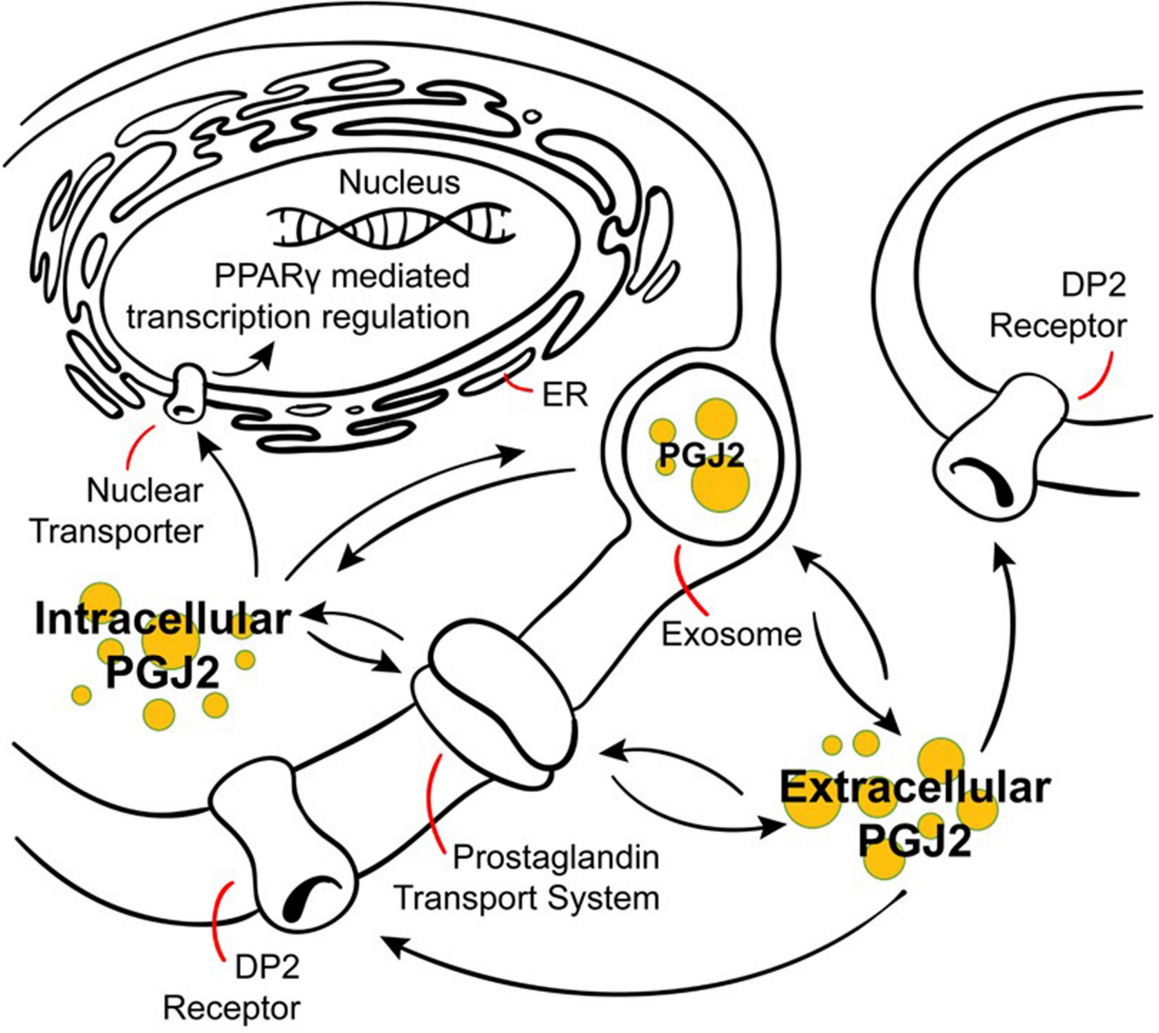
**Modes of action of J2 prostaglandins**. PGJ2 and its metabolites exit the cell via diffusion or poorly defined transporters, and can enter cells or the nucleus via active transporters at the plasma or nuclear membranes. PGJ2 and its metabolites exert their actions by different mechanisms. They can bind to the DP2 receptor on the plasma membrane or to the peroxisomal proliferator activator receptor (PPARγ) at the nuclear membrane. Trafficking of PGJ2 and its metabolites in and out of cells can also occur via exosomes.

Despite their lipid nature, prostaglandins are charged anions thus have low intrinsic permeability across the plasma membrane, but they cross it twice (Figure 4): once following their synthesis as they are released from the cytoplasm into the extracellular environment (efflux), and then again when they undergo reuptake into the cytoplasm (influx), a process that mimics neurotransmitter reuptake (Schuster, 1998; Chan et al., 2002; Chi et al., 2014). Prostaglandin efflux is mediated (a) by diffusion driven by pH and the membrane potential, and (b) by the action of transporters such as multidrug resistance-associated proteins (MRPs) and prostaglandin transporters (PGTs) (Schuster, 2002; Ohkura et al., 2014). Prostaglandin influx is mediated by PGTs as well (Schuster, 2002; Chi et al., 2006). Overall, prostaglandin transport requires further investigation. A well-characterized PGT belongs to the family of the 12-transmembrane organic anion transporting polypeptides (OATPs), and mediates the influx of prostaglandins only (Chi et al., 2006; Chi and Schuster, 2010). PGT mediated influx is a required step for prostaglandin metabolism (Nomura et al., 2004), which occurs intracellularly. PGD2 is a substrate for PGT (Itoh et al., 1996) and its metabolism to PGJ2 highly likely involves PGT mediated uptake. Interestingly, this PGT is a lactate/prostaglandin exchanger in which prostaglandin influx varies with lactate levels, so that cells engaged in glycolysis thus producing high levels of lactate, are energetically poised to uptake prostaglandins via this PGT (Chan et al., 2002; Banu et al., 2003). This PGT is present in many tissues including the brain (Kanai et al., 1995; Lu et al., 1996).

Prostaglandins also can be transferred from cell to cell via exosomes, which are extracellular bioactive vesicles released from multivesicular bodies that mediate intercellular signaling (Subra et al., 2010). As such, exosomes are considered intercellular “signalosomes” as they carry arachidonic acid, phospholipases (A2 and D2), COX-1 and 2, and a whole set of prostaglandins, including PGD2, E2, and J2 (Subra et al., 2010). In fact, the concentrations of these prostaglandins within exosomes is in the micromolar range, thus at concentrations capable of triggering prostaglandin-dependent biological effects (Subra et al., 2010). Moreover, exosome internalization by neighboring cells is considered a mechanism for prostaglandins to reach their intracellular targets (Subra et al., 2010). Exosomes are released from and taken up by neurons in a synaptic activity-dependent manner that is also regulated by calcium (Lachenal et al., 2011; Perez-Gonzalez et al., 2012; Chivet et al., 2013; Morel et al., 2013). Furthermore, exosomes have recently been considered to be propagation vehicles for spreading of toxic proteins (Bellingham et al., 2012) as well as prostaglandins, such as neurotoxic PGJ2, and could thus play a significant role in the spread of pathology in a variety of neurodegenerative disorders (Schneider and Simons, 2013).

As they are unstable, prostaglandins exert their effects near their sites of synthesis thus acting as autocrine or paracrine ligands (Scher and Pillinger, 2005). The efflux of newly synthesized prostaglandins mediates their biological actions through their cell surface receptors, and prostaglandin influx from the extracellular milieu mediates their action through specific nuclear receptors or their inactivation (Banu et al., 2003). Prostaglandin inactivation in the cytoplasm is carried out by the enzyme NAD(+)-linked 15 hydroxyprostaglandin dehydrogenase (15-PGDH); its expression and that of COX-2 are reciprocally regulated in cancer, thus both enzymes control the cellular levels of prostaglandins by opposing means (Tai et al., 2006; Tai, 2011).

The effects of J2 prostaglandins are mediated by at least three different means.

### G protein-coupled receptors (GPCR, Figure 4)

In general, the prostaglandin GPCRs are present not only at the plasma membrane (Ricciotti and FitzGerald, 2011) but also at the nuclear membrane, thus providing for intracrine (intracellular) signaling (Zhu et al., 2006). PGD2, the precursor of PGJ2, binds to the DP1 and DP2 receptors (Urade and Eguchi, 2002). While the activation of the DP1 receptor is coupled to the G protein G*s*, resulting in increased cAMP levels, activation of the DP2 receptor and its coupling to G*i* decreases cAMP levels, and increases intracellular calcium (Hata and Breyer, 2004). J2 prostaglandins bind to DP1 and DP2, however they have a higher affinity for DP2 (as much as 100-fold) and bind to it with an affinity similar to PGD2, i.e., in the nanomolar range (Monneret et al., 2002; Pettipher et al., 2007). DP2 activation was shown to potentiate neuronal injury in hippocampal neuronal cultures and organotypic slices, while DP1 activation is neuroprotective (Liang et al., 2005, 2007).

### Nuclear receptors (Figure 4)

15d-PGJ2 and Δ^12^-PGJ2 are endogenous ligands for the nuclear peroxisomal proliferator activator receptor (PPARγ), to which they bind with high affinity (Gilroy, 2010; Paulitschke et al., 2012), although this remains controversial (Ricciotti and FitzGerald, 2011). PPARγ plays a major role in the regulation of adipogenesis, glucose homeostasis, cellular differentiation, apoptosis and inflammation (Qi et al., 2010). PPARγ agonists promote neuroprotection in models of stroke, AD, HD, PD, MS, and spinal cord injury, via anti-inflammatory or antioxidant-dependent mechanisms (Kapadia et al., 2008; Kiaei, 2008).

J2 prostaglandins also act through PPARγ-independent mechanisms including activation of the MAPK and JNK pathways (Wilmer et al., 2001; Li et al., 2004a), stabilization of the transcription factor Nrf2 via its interaction with Keap1 (Itoh et al., 2004; Kaspar et al., 2009; Haskew-Layton et al., 2013), and inhibition of the NFκB pathway (Rossi et al., 2000; Straus et al., 2000). This may account for the different effects of 15d-PGJ2 and other PPAR ligands (Scher and Pillinger, 2005).

### Direct interaction with intracellular proteins causing a specific post-translational modification (Figure 5)

**Figure 5:**
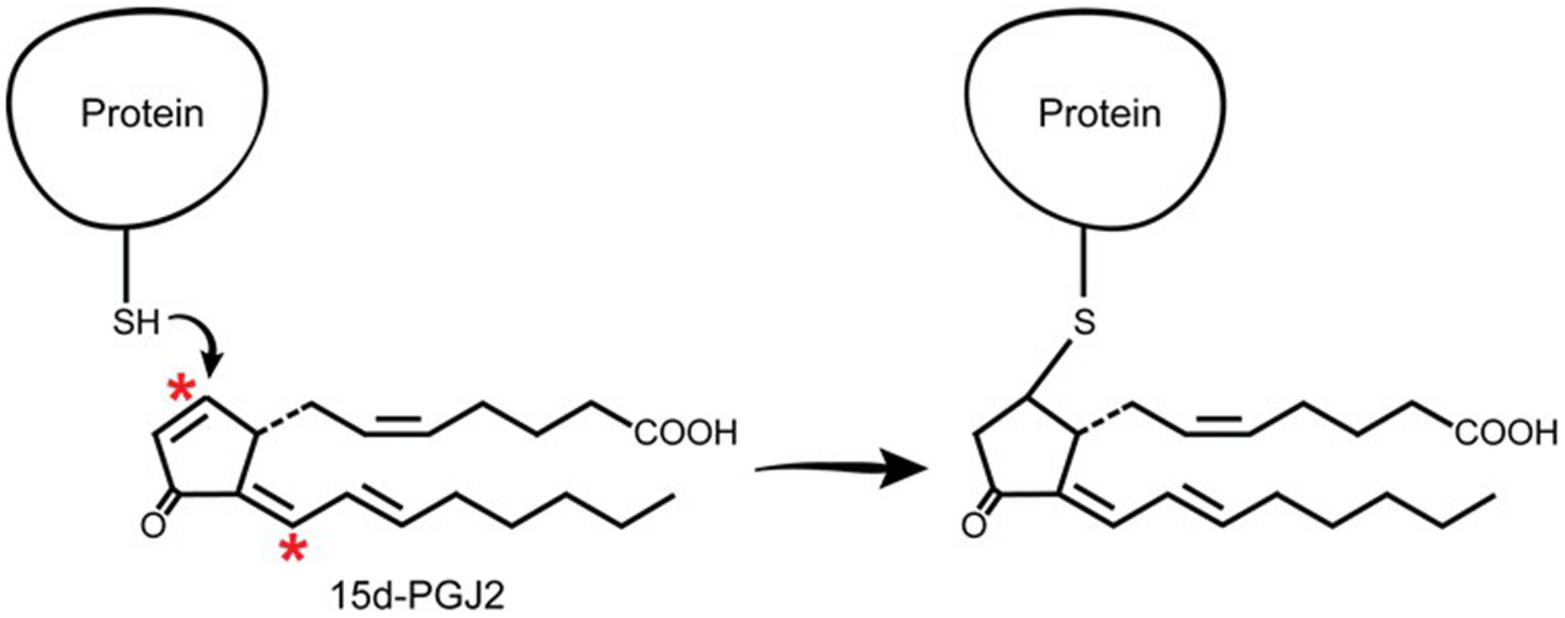
**J2 prostaglandins interact directly with cellular proteins**. J2 prostaglandins (shown for 15d-PGJ2) covalently modify selective proteins through Michael addition. Their α,β-unsaturated carbonyl groups (asterisks) react with free sulfhydryls (SH) in cysteines on glutathione and cellular proteins.

J2 prostaglandins are unique among the prostaglandin family in that they have α,β-unsaturated carbonyl groups (*asterisks*, Figure 5), promoting Michael addition reactions with free sulfhydryl groups of cysteines in glutathione and cellular proteins (Straus and Glass, 2001). These cyclopentenone PGs covalently modify several proteins, including the p50 subunit of NFκB, which may explain its anti-inflammatory effects (Cernuda-Morollon et al., 2001). They also modify thioredoxin reductase, an enzyme that protects against oxidative damage (Moos et al., 2003) and activate Ras, a small GTPase oncogene known to activate Erk signaling pathways (Oliva et al., 2003).

Electrophile binding to key protein cysteine(s) by endogenous compounds such as PGJ2 is regarded as playing an important role in determining whether neurons will live or die (Satoh and Lipton, 2007). In fact, with neuronal cultures, we established that PGJ2 was by far the most neurotoxic of four prostaglandins tested, including PGA1, D2, E2, and J2, with PGE2 being the least neurotoxic of the four under the conditions tested (Li et al., 2004b). The response to J2 prostaglandins is different from that of agonists that do not form covalent adducts with proteins. A slow steady stream-like release of J2 prostaglandins as a result of chronic neuroinflammation could be cumulative, leading overtime to accumulation of covalent PGJ2-protein adducts until they reach a toxic threshold. Thus, covalent protein modification in the brain by highly reactive electrophiles such as J2 prostaglandins, represents a novel pathologic post-translational change (Higdon et al., 2012b) and could play a critical role in progressive neurodegeneration.

## J2 prostaglandin: potential transition to chronic inflammation and pathology spreading

**Figure 6:**
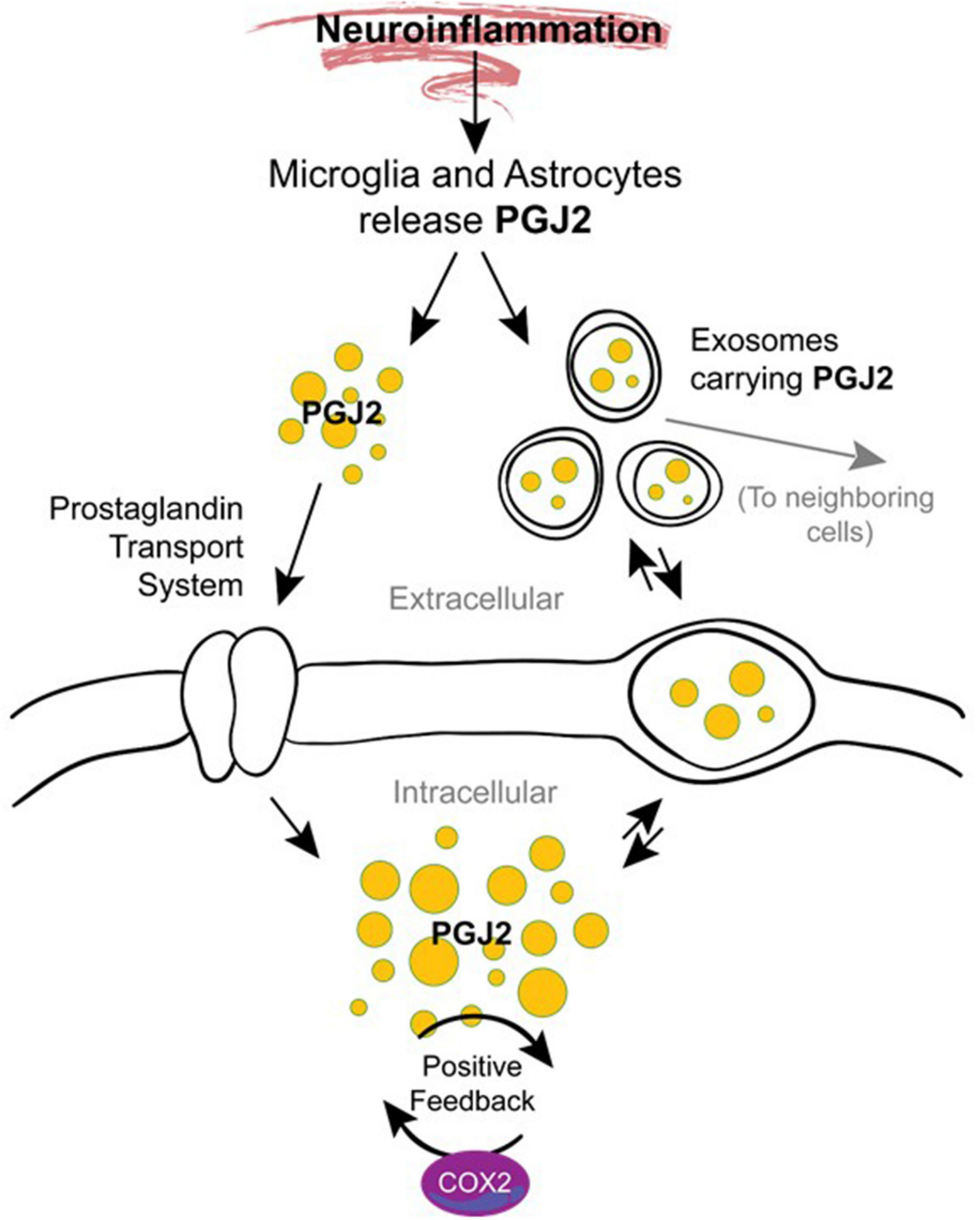
**Potential mechanisms by which J2 prostaglandins promote neurodegeneration**. During neuroinflammation PGJ2 (and its metabolites) are released from activated microglia and astrocytes. Free or exosome enclosed PGJ2 mediates the spread of neurodamage within the brain via intercellular uptake. PGJ2 also increases the levels of COX-2, thus activating a positive feedback loop that could mediate the transition from acute to chronic inflammation.

The mechanisms underlying the transition from acute to chronic inflammation are poorly understood. One hypothesis supported by animal studies is that in addition to their role in mediating acute inflammation, prostaglandins also function in the transition and maintenance of chronic inflammation, culminating in long-lasting effects (Aoki and Narumiya, 2012). Prostaglandins accomplish this by amplifying cytokine signaling, up-regulating COX-2 (Figure 6), inducing chemokines, and recruiting inflammatory cells such as macrophages (Aoki and Narumiya, 2012). We and others demonstrated that J2 prostaglandins induce COX-2 up-regulation in cancer cells (Kim et al., 2008; Kitz et al., 2011) and neuronal cells (Li et al., 2004a) and this event can be driven by MAPK activation (Li et al., 2004a; Kitz et al., 2011) or Akt/AP-1 activation (Kim et al., 2008).

It is also possible that J2 prostaglandins mediate the spread of neurodamage within the brain via exosomes (Figure 6), as discussed above under “Modes of action of J2 prostaglandins.” This is important because exosomes were recently considered propagation vehicles for spreading neuropathology in a range of neurodegenerative diseases including AD, PD, and HD (Schneider and Simons, 2013). *In vitro* studies demonstrate that exosomes are released from neurons in a synaptic activity-dependent manner regulated by calcium (Lachenal et al., 2011). This later aspect of the regulation of the exosome-release by calcium could be particularly relevant to J2 prostaglandins. They are potent agonists (EC_50_ of ~10 nM) of the DP2 receptor (Monneret et al., 2002), which signals through elevation of intracellular calcium and reduction in intracellular cAMP (Pettipher et al., 2007), thus J2 prostaglandins could induce exosome-release.

These findings support the notion that targeting prostaglandin signaling for therapeutics represents a highly innovative strategy to prevent/block chronic neuroinflammation and disease progression.

## J2 prostaglandin targets: UPP and mitochondria

**Figure 7:**
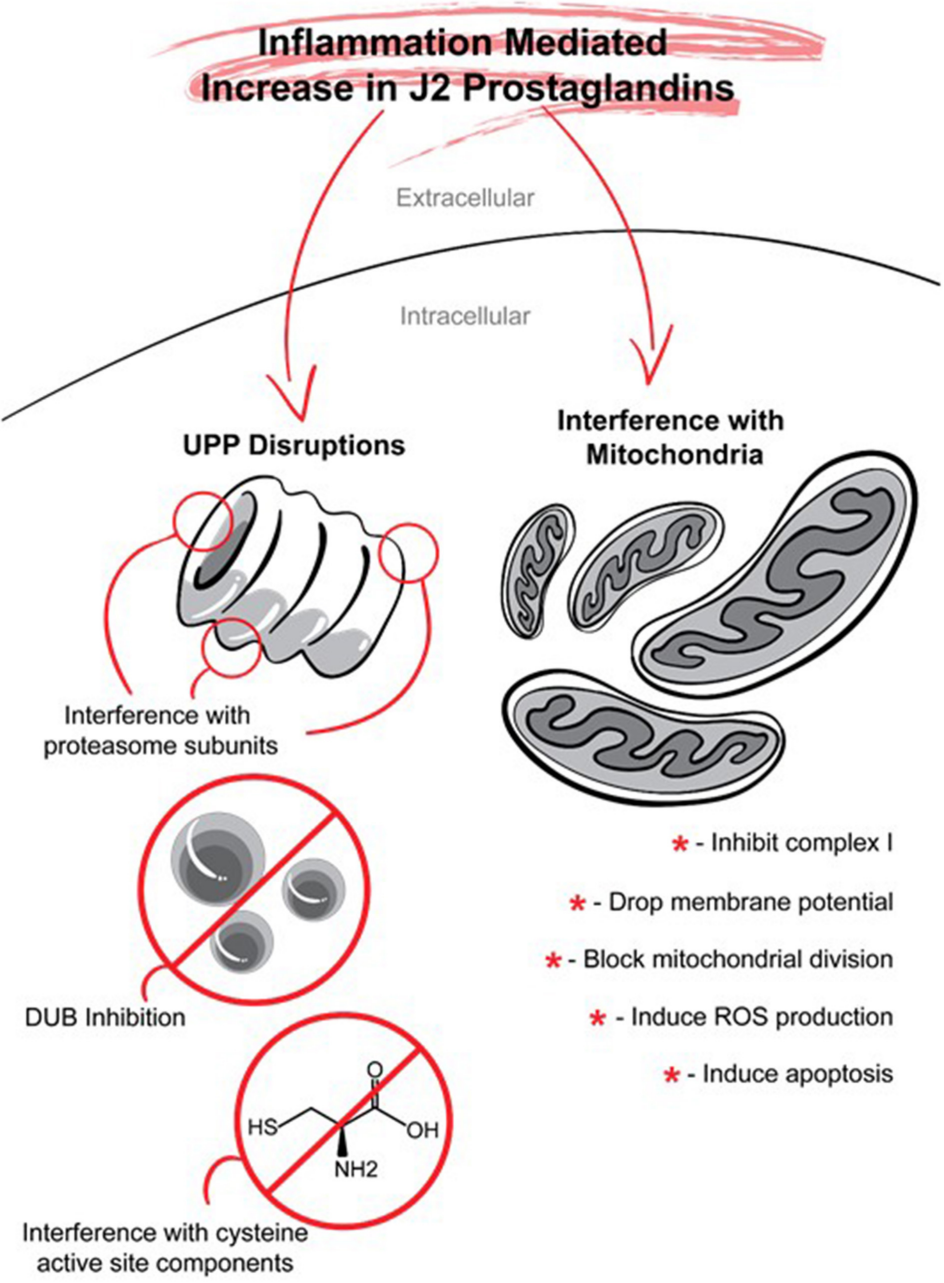
**J2 prostaglandins target the ubiquitin proteasome pathway (UPP) and mitochondria**. J2 prostaglandins affect the UPP by: (1) impairing the 26S proteasome by inducing oxidation of proteasome subunits, or promoting its disassembly, (2) inhibiting de-ubiquitinating enzymes (DUBs), and (3) covalently modifying specific active site cysteines on UPP components such as E1 activating enzymes, E2 conjugating enzymes, and some E3 ligases. J2 prostaglandins can also inhibit mitochondrial function by: (1) inhibiting NADH-ubiquinone reductase in complex I, (2) reducing membrane potential, (3) blocking fission, and (4) inducing the generation of reactive oxygen species (ROS) and apoptosis.

COX-2 neurotoxicity seems to be mediated by PGD2 but not by PGE2 (Liang et al., 2005). PGD2 is the most abundant prostaglandin in the brain (Abdel-Halim et al., 1977; Narumiya et al., 1982; Hertting and Seregi, 1989). For example, in young rats (16–18 post-natal) subjected to a 12-min asphyxial cardiac arrest, the brain levels of PGE2 assessed by UPLC–MS/MS were ~35.5 pmol/g of tissue, while those of PGD2 were at least 26 fold higher, reaching ~937 pmol/g of tissue (Shaik et al., 2014). PGD2 elicits its cytotoxicity via its bioactive metabolites J2 prostaglandins (Liu et al., 2013b). In contrast to other reviews (Musiek et al., 2005; Uchida and Shibata, 2008; Scher and Pillinger, 2009; Surh et al., 2011; Oeste and Perez-Sala, 2014), our review addresses in detail the effects of J2 prostaglandins on two targets that play key roles in the neurodegenerative process, namely, the ubiquitin-proteasome pathway and mitochondrial function (Figure 7).

### Ubiquitin-proteasome pathway (UPP, Figure 7)

It is well-established that in neuronal cells J2 prostaglandins trigger the accumulation/aggregation of ubiquitinated proteins (see for example Li et al., 2004b; Ogburn and Figueiredo-Pereira, 2006; Liu et al., 2011, 2013a). This protein accumulation/aggregation can be mediated by at least two mechanisms shown to be affected by J2 prostaglandins.

#### Inhibition of the 26S proteasome

We and others demonstrated that J2 prostaglandins impair the 26S proteasome (Shibata et al., 2003; Ishii and Uchida, 2004; Wang et al., 2006; Koharudin et al., 2010). In neuronal cells, these prostaglandins induce the oxidation of at least one proteasome subunit, i.e., S6 ATPase (Rpt5), which seems to be one of the proteasome subunits most vulnerable to protein carbonylation (Ishii et al., 2005). PGJ2 also promotes dissociation of the 20S core particle from the 19S regulatory particle (Wang et al., 2006), resembling the effects of agents that induce oxidative stress (Aiken et al., 2011). The effect of J2 prostaglandins on the proteasome are attributed to their electrophilic properties, since a 15d-PGJ2 analog that lacks the double bond in the cyclopentenone ring failed to inhibit the proteasome (Shibata et al., 2003). Furthermore, 15d-PGJ2/proteasome conjugates were detected in neuronal cells treated with biotinylated 15d-PGJ2 (Shibata et al., 2003).

These data indicate that one of the effects of inflammation mediated by J2 prostaglandin is proteasome inhibition. Conversely, proteasome inhibition suppresses inflammation (Bi et al., 2012). It was recently shown that the mechanisms by which the antibiotic rifampicin suppresses microglia activation upon LPS-treatment, is by downregulating the Rpt1 (MSS1) proteasome subunit (Bi et al., 2012). Likewise, downregulation of the proteasome subunit Rpn9 (PSMD13) by siRNA suppresses microglial activation and diminishes the production of inflammation associated factors such as nitric oxide synthase, nitric oxide, cyclooxygenase-2, and prostaglandin E2 (Bi et al., 2014).

A close relation between proteasome and inflammation is further supported by the finding that cyclooxygenases 1 and 2 are turned over by the 26S proteasome (Rockwell et al., 2000; Yazaki et al., 2012) via the endoplasmic reticulum-associated degradation (ERAD) pathway (Mbonye et al., 2006, 2008). Proteasomal-mediated turnover of COX-2 is regulated by the G protein-coupled receptor prostaglandin E1 (EP1 for PGE2) independently of receptor activation (Haddad et al., 2012). EP1 is present both at the plasma membrane and at the inner and outer membrane of the nuclear envelop (Bhattacharya et al., 1998). EP1 may act as a scaffold for an E3 ligase that ubiquitinates COX-2 (Haddad et al., 2012). In addition to the cyclooxygenases, prostaglandin synthases are also turned over by the proteasome. As such, increases in intracellular calcium result in the rapid proteasomal-mediated degradation of the PGD2 prostaglandin synthase H-PGDS in human megakaryocytic cells (Yazaki et al., 2012). Degradation of other prostaglandin synthases by the proteasome remains to be investigated.

#### Inhibition of de-ubiquitinating enzymes (DUBs)

The UPP and autophagy play a critical role in protein quality control and thus have attracted special attention for drug development (Edelmann et al., 2011). In regard to the UPP, which is the focus of this review, the initial effort was directed toward proteasome inhibitors, although not particularly for neurological conditions (Ristic et al., 2014). However, proteasome inhibition can be quite unspecific and thus may lead to a range of undesirable side effects. De-ubiquitinating enzymes known as DUBs are relevant UPP targets upstream of the proteasome and are the focus of recent attention for drug development. Several DUBs have been targeted for cancer treatment (Crosas, 2014), and one for neurological conditions (USP14) (Lee et al., 2010). At least one hundred (if not more) DUBs from five different gene families are present in humans, and four of these families are thiol proteases and one is a metalloprotease (Eletr and Wilkinson, 2014). These DUBs carry-out different functions, some of which involve processing newly translated ubiquitin (Ub) to provide monomers for conjugation and chain formation, or trimming mono-Ub from the distal end of a poly-Ub chain, or disassembling poly-Ub chains, or removing poly-Ub chains from their substrates (Clague et al., 2013). All together these functions provide a means for DUBs to regulate “where, when, and why” ubiquitinated substrates are degraded by the 26S proteasome (Ristic et al., 2014).

We and others showed that J2 prostaglandins inhibit some of the thiol DUBs including UCH-L1 and UCH-L3 (Mullally et al., 2001; Li et al., 2004b; Liu et al., 2011). In particular, UCH-L1 is predicted to be one of the most abundant proteins in the brain reaching 1–2% of the total protein content (Wilkinson et al., 1992), and its activity is highly diminished by J2 prostaglandins (Li et al., 2004b; Koharudin et al., 2010). These prostaglandins trigger UCH-L1 unfolding and aggregation by forming a covalent adduct with a single thiol group on Cys 152 (Koharudin et al., 2010). UCH-L1 plays an important role in aging and neurodegenerative disorders such as AD and PD, thus its alterations by J2 prostaglandins are highly relevant to the neurodegenerative process as discussed below under “neurodegenerative disorders” and reviewed in (Ristic et al., 2014).

#### Alteration of other UPP components that have cysteines at the active site

Other components of the UPP, such as E1 activating enzymes, E2 conjugating enzymes, and some E3 ligases also have active site cysteines (Metzger et al., 2014). Whether J2 prostaglandins affect these and/or other thiol DUBs remains to be investigated. Protein modification by J2 prostaglandins seems to be highly selective where only a specific set of cysteine residues are vulnerable to covalent modification and this event occurs independently of protein thiol content (Higdon et al., 2012a; Vasil'ev et al., 2014). Of the several hundred cellular proteins with potentially reactive thiols, only ~10% form covalent adducts with J2 prostaglandins, and are considered members of the “electrophile-responsive proteome” (Ceaser et al., 2004). Although J2 prostaglandins interact with selective targets, they can covalently modify a wide variety of intracellular proteins. Proteomic approaches established that J2 prostaglandins covalently bind to specific sites within the plasma membrane, nuclear and cytosolic subcellular fractions (Yamamoto et al., 2011). At least eleven plasma membrane proteins were identified as binding biotinylated 15d-PGJ2 and they were distributed into three functional groups: glycolytic enzymes, molecular chaperones, and cytoskeletal proteins (Yamamoto et al., 2011). Furthermore, J2 prostaglandin binding alters protein catalysis, binding, structural function, and transport (Marcone and Fitzgerald, 2013; Oeste and Perez-Sala, 2014).

### Mitochondria (Figure 7)

J2 prostaglandins inhibit mitochondrial function leading to oxidative stress and apoptosis (Kondo et al., 2001; Lee et al., 2009; Paulitschke et al., 2012). These prostaglandins inhibit the activity of the enzyme NADH-ubiquinone reductase from mitochondrial respiratory complex I, most likely by adduct formation (Martinez et al., 2005). J2 prostaglandins also induce the generation of reactive oxygen species (ROS), trigger a drop in membrane potential (Pignatelli et al., 2005; Gutierrez et al., 2006), interact with the cytoskeleton (Stamatakis et al., 2006) and block mitochondrial division through covalent modification of dynamin-related protein 1 (Drp1) which regulates mitochondrial fission (Mishra et al., 2010). A mitochondrial targeted analog of 15d-PGJ2 (mito-15d-PGJ2) was more efficient than 15d-PGJ2 at inducing apoptosis, was less potent at up-regulating Keap1-dependent antioxidant expression of HO-1 and GSH, and caused profound defects in mitochondrial bioenergetics and mitochondrial membrane depolarization (Diers et al., 2010). This interesting approach that involved specifically targeting 15d-PGJ2 to mitochondria by conjugation to a lipophilic cation, demonstrates the feasibility of manipulating its biological effects (Diers et al., 2010).

### UPP/mitochondria interaction

UPP and mitochondrial function are inherently connected, and since J2 prostaglandins affect both processes we will discuss this interaction. On the one hand the UPP degrades various mitochondrial proteins contributing to mitochondrial quality control (Taylor and Rutter, 2011). Mitochondrial proteins that are UPP substrates include (a) damaged and/or misfolded nuclear encoded proteins that are destined for import into mitochondria, (b) defective proteins at the outer mitochondrial membrane (OMM) extracted by p97 and delivered to the proteasome, and (c) non-OMM proteins, although the mechanistic details of how these proteins in the inner compartments retrotranslocate to the OMM remains poorly defined (Shanbhag et al., 2012). In addition, both the ubiquitin ligase parkin (Narendra and Youle, 2011) and the de-ubiquitinating enzyme USP30 (Bingol et al., 2014) seem to regulate mitochondrial turnover via mitophagy. The PINK1-Parkin pathway proposed to promote mitophagy remains controversial in neurons (Exner et al., 2012).

On the other hand, mitochondria provide ATP for protein ubiquitination and for 26S proteasome function (Livnat-Levanon and Glickman, 2011). E1 activity, the first step of the ubiquitination cascade, requires ATP for formation of a thiol ester adduct with ubiquitin (Haas et al., 1982; Schulman and Harper, 2009). If E1 activity is impaired all protein ubiquitination should be diminished. Moreover, the degradation of proteins by the 26S proteasome is highly dependent on ATP binding and hydrolysis (Liu et al., 2006).

It is postulated that in neurons even a modest restriction of ATP production by mitochondria far outweighs the negligible effects of ROS, although the underlying mechanisms are not clearly understood (Nicholls, 2008). In a recent study with neurons (Huang et al., 2013), we demonstrated that low ATP levels caused by mitochondrial dysfunction, correlated with impairment of the UPP: there was a decline in E1 and 26S proteasome activities with a concomitant rise in 20S proteasomes. This decline in UPP function occurred upon acute and long-term mitochondrial impairment. Notably, upon energy depletion, calpain activation led to the selective cleavage of Rpn10, a 26S proteasome subunit, without affecting other proteasome subunits tested. Rpn10 cleavage combined with ATP depletion, contributed to the decline in 26S proteasome function. We postulated that upon mitochondrial dysfunction, ATP-depletion and calpain activation contribute to the demise of protein turnover by the UPP in favor of unregulated and energy-independent protein degradation by 20S proteasomes. This adaptive response to energy deficiency may be suitable for short-term periods to promote degradation of randomly unfolded oxidized proteins. However, if chronic it can lead to neurodegeneration, as regulated protein degradation by the UPP is essential for neuronal survival (Huang et al., 2013).

### Complex effects of J2 prostaglandins

Overall, the role of J2 prostaglandins in inflammation is complex (Harris et al., 2002; Wall et al., 2012). On the one hand, they have emerged as key anti-inflammatory agents as they inhibit the production of pro-inflammatory mediators such as iNOS, TNFα, and IL1β, suppress microglia and astrocyte activation and induce apoptosis (Eucker et al., 2004; Giri et al., 2004; Mrak and Landreth, 2004). On the other hand, J2 prostaglandins are pro-inflammatory agents. They stimulate the production of pro-inflammatory mediators such as IL8 and activate MAPK (Meade et al., 1999; Zhang et al., 2001). Furthermore, 15d-PGJ2 seems to play a role in the regulation of human autoimmune diseases and to inhibit inflammation in models of arthritis, ischemia-reperfusion injury, inflammatory bowel disease, lupus nephritis, and AD (Scher and Pillinger, 2009).

J2 prostaglandins also display both protective and destructive effects. Their biological activities include antiviral and antitumoral effects, modulation of the heat shock response, induction of oxidative stress, and apoptosis (Uchida and Shibata, 2008), as well as up-regulation of the death receptor 5 (DR5), sensitization to TRAIL-induced cytotoxicity, and caspase 8 activation (Kondo et al., 2002; Nakata et al., 2006; Su et al., 2008; Metcalfe et al., 2012). Although their anti-proliferative and pro-apoptotic effects are most frequently described, J2 prostaglandins also induce the proliferation of different forms of cancer cells when used at nanomolar to low micromolar concentrations (Oliva et al., 2003).

The conflicting effects of J2 prostaglandins most likely depend on their intracellular targets and downstream pathways which can be dose- and cell-type dependent (Servidei et al., 2004). J2 prostaglandins may exert some of their anti- or pro-inflammatory as well as anti- or pro-survival effects through PPARγ-dependent mechanisms. However, these prostaglandins also exert their actions through PPARγ-independent pathways as discussed above under “modes of action of J2 prostaglandins.” As such, it is known that specific PPARγ ligands, such as pioglitazone, do not replicate all of the effects attributed to J2 prostaglandins. Identifying the mechanism by which J2 prostaglandins exert their neurotoxic effects could lead to new strategies to prevent and/or delay neurodegeneration linked to inflammation.

## J2 postaglandins and neurodegenerative disorders (Table 2)

Chronic neuroinflammation is recognized as a primary mechanism involved in neurodegenerative diseases such as AD, PD, and ALS (Liu and Hong, 2003; Glass et al., 2010; Cudaback et al., 2014; Mosher and Wyss-Coray, 2014). Moreover, UPP and mitochondrial dysfunction as interdependent cellular events (Livnat-Levanon and Glickman, 2011; Taylor and Rutter, 2011) are both impaired in many neurodegenerative disorders (Lin and Beal, 2006; Paul, 2008). However, the underlying mechanisms that bring about and/or maintain these malfunctions are unknown. A self-perpetuating cycle of inflammatory processes involving brain immune cells (microglia and astrocytes) could drive the slow progression of the neurodegenerative process, leading to dysfunction in protein degradation by the UPP and ATP production by mitochondria. Preventing/arresting this self-perpetuating inflammatory cycle is a very promising neuroprotective strategy for these disorders (Lima et al., 2012). We will focus on a review of studies showing that J2 prostaglandins are associated with AD, PD, and ALS, as well as stroke, TBI and Krabbe disease (Table 2).

**Table 2:** **J2 postaglandins and neurodegenerative disorders**.

**Disease**	**PGD2/PGJ2 pathway**	**Effects/Findings**	**References**
**Alzheimer**	**PGD2**	↑ Levels in frontal cortex of AD patients	Iwamoto et al., 1989; Yagami, 2006
	**PGJ2**	↓ UPP (UCH-L1 and proteasome) function, relevant to AD and other neurodegenerative disorders	Li et al., 2004b; Choi et al., 2004; Upadhya and Hegde, 2007; Oddo, 2008; Liu et al., 2011
		↑ Caspase-dependent TAU cleavage	Arnaud et al., 2009; Metcalfe et al., 2012
		↑ Ub protein and TAU aggregation	
	**H-PGDS**	↑ Levels in AD patients and Tg2576 mouse (in astrocytes and microglia within senile plaques)	Mohri et al., 2007
	**L-PGDS**	↑ Levels in AD patients and Tg2576 mouse (within senile plaques)	Kanekiyo et al., 2007
			Lee et al., 2012
		↑ Binds Ap monomers, prevents aggregation	
		↓ Promotes migration and morphological changes of microglia and astrocytes via MARCKS protein	
	**DPI**	↑ Levels in AD patients and Tg2576 mouse (in astrocytes and microglia within senile plaques)	Mohri et al., 2007
	**PGT**	↓ Levels in AD patient brain homogenates	Choi et al., 2008
**Parkinson**	**PGD2**	α-synuclein modulates arachidonic acid metabolism and downstream PGD2/PGJ2 production	Castagnet et al., 2005; Golovko et al., 2006; Golovko and Murphy, 2008
	**PGJ2**	PGJ2-induced mouse model exhibits slow-onset PD-like pathology	Pierre et al., 2009; Shivers et al., 2014
		Optimal for testing diagnostic tools (such as PET) and therapeutic interventions for neurons and microglia	
	**L-PGDS**	Isoform changes in CSF of PD patients Potential PD biomarker	Harrington et al., 2006
**ALS**	**PGJ2**	15d-PGJ2 accumulates in spinal motor neurons of ALS patients	Kondo et al., 2002; Zhang et al., 2010
	**DPI**	Blocking DP1 as a therapeutic strategy	Di Giorgio et al., 2008; de Boer et al., 2014
**Stroke**	**PGJ2**	↑ Levels in the brains of rodent models of cardiac arrest and stroke	Liu et al., 2011, 2013a,b; Shaik et al., 2014
**TBI**	**PGJ2**	↑ Levels in the brains of rodent models of TBI PPARy ligands protective or deleterious?	Kunz et al., 2002; Hickey et al., 2007 Qi et al., 2010; Surh et al., 2011
**Krabbe**	**H-PGDS DPI**	KO of the H-PGDS or the DP1 receptor, or inhibiting H-PGDS with HQL-79 in the *twitcher* mouse model is protective	Mohri et al., 2006; Bosetti, 2007; Palumbo and Bosetti, 2013

### Alzheimer disease

PGD2 levels were found to be significantly increased in the frontal cortex of AD patients compared to age matched controls (Iwamoto et al., 1989; Yagami, 2006). In AD patients and in Tg2576 mice, a well-established AD model (Hsiao et al., 1996), the levels of the PGD2 synthase H-PGDS and the PGD2 receptor DP1 were found to be selectively up-regulated in microglia and astrocytes within senile plaques (Mohri et al., 2007). These results support the notion that PGD2 acts as a mediator of plaque associated inflammation in the AD brain and they could also explain the pharmacologic mechanisms underlying the favorable response of patients with AD to non-steroidal anti-inflammatory drugs (Mohri et al., 2007).

The other PGD2 synthase L-PGDS, which is one of the most abundant CSF proteins produced in the brain, was localized in amyloid plaques in both AD patients and Tg2576 mice (Kanekiyo et al., 2007). Secreted L-PGDS in the CSF has a dual function: it increases CSF-PGD2 levels (Scher and Pillinger, 2005) and also acts as a lipophilic-ligand carrier (Urade and Hayaishi, 2000). L-PGDS was found to bind Aβ monomers and prevent Aβ aggregation, suggesting that L-PGDS is a major Aβ chaperone and disruption of this function could be related to the onset and progression of AD (Kanekiyo et al., 2007).

L-PGDS and PGD2 also promote migration and morphological changes of microglia and astrocytes that resemble those exhibited under reactive gliosis (Lee et al., 2012). This L-PGDS function is mediated by its interaction with myristoylated alanine-rich protein kinase C substrate (MARCKS), which in turn activates the AKT/Rho/JNK pathway (Lee et al., 2012). MARCKS is a plasma membrane resident abundant in the nervous system, and that depending on its phosphorylation by PKC acts as an actin cross-linker to regulate cellular adhesion and spreading, migration, proliferation, and fusion through its interaction with the cytoskeleton (Arbuzova et al., 2002). MARCKS also plays a role in the maintenance of dendritic spines and contributes to PKC-dependent morphological plasticity in hippocampal neurons (Calabrese and Halpain, 2005). Together these results support an essential role for L-PGDS in the regulation of glial cell migration and morphology, and perhaps neuronal plasticity, within the CNS (Lee et al., 2012).

The transport of prostaglandins across cell and organelle membranes involves, among others, a prostaglandin transporter (PGT) (Kanai et al., 1995). Immunohistochemical and immunofluorescent analyses of this PGT in human brains showed its localization in neurons, microglia, and astrocytes in all brain tissues assessed (Choi et al., 2008). In addition, PGT levels were lower in AD than in age-matched control brain homogenates, suggesting that prostaglandins might not be cleared at the normal rate in AD brains (Choi et al., 2008).

In relation to J2 prostaglandins, the finding that they impair the UPP is highly relevant to AD (Selkoe, 2004; Shaw et al., 2007). Defective proteasome activity is linked to the early phase of AD characterized by synaptic dysfunction, as well as to late AD stages linked to accumulation and aggregation of ubiquitinated (Ub)-proteins in both senile plaques and neurofibrillary tangles (Upadhya and Hegde, 2007; Oddo, 2008). Moreover, J2 prostaglandins inhibit the de-ubiquitinating enzyme UCH-L1 (Li et al., 2004b; Liu et al., 2011), which is down-regulated in AD brains; UCH-L1 down-regulation is inversely proportional to the number of neurofibrillary tangles (Choi et al., 2004).

Besides the effects on the UPP, we showed in rat primary cerebral cortical cultures that PGJ2 induced accumulation of Ub-proteins, caspase-activation, TAU cleavage at Asp421, and neuritic dystrophy (Arnaud et al., 2009; Metcalfe et al., 2012). TAU cleavage at Asp421 was identified as an early event in AD tangle pathology (Gamblin et al., 2003; Rissman et al., 2004; de Calignon et al., 2010). In summary, J2 prostaglandins mimic many pathological processes observed in AD.

### Parkinson disease (PD)

Up-regulation of PGD2 or J2 prostaglandins in PD patients has not been addressed yet, but there is ample evidence linking PGD2, the precursor of PGJ2, to PD. Changes in PGD2 levels occurring in PD brains will lead to parallel changes in the highly reactive cyclopentenone J2 prostaglandins because PGD2 is unstable and is spontaneously metabolized to J2 prostaglandins. It was calculated that the half-life of PGD2 in the brain is 1.1 min (Suzuki et al., 1986).

Significant changes in L-PGDS isoforms were detected in the CSF of at least 20 idiopathic PD patients compared to 100 controls (Harrington et al., 2006). These alterations reflected up/down regulation of L-PGDS isoforms likely (a) to represent pathology at the cellular level expected to impact prostaglandin production, and (b) to correlate with disease symptoms (Harrington et al., 2006). It was speculated that these altered isoforms could be candidate diagnostic PD biomarkers and may have predictive value (Harrington et al., 2006).

A number of studies suggest that α-synuclein plays a role in brain fatty acid metabolism including arachidonic acid, through modulation of ER-localized acyl-CoA synthetase activity (Castagnet et al., 2005; Golovko et al., 2006). Acyl-CoA synthetase is an enzyme that converts fatty acids to acyl-coA for subsequent beta oxidation. In α-synuclein KO mice, exogenous addition of wild-type mouse or human α-synuclein restored acyl-CoA synthetase activity, while mutant (A30P, E46K, and A53T) forms of α-synuclein did not (Golovko et al., 2006). In addition, the levels of several prostaglandins in brains following a 30 s global ischemia were compared in wild type vs. α-synuclein KO mice (Golovko and Murphy, 2008). Among all prostaglandins assayed (E2, D2, F2α, TxB2, and 6-ketoF1α) PGD2 showed the greatest increase (two-fold) in the α-synuclein KO mice relative to the wild type. The levels of PGD2 in brains of α-synuclein KO mice reached ~35 ng/g following the 30 s global ischemia. Under normal physiological conditions, α-synuclein ablation had no effect. Together these studies suggest that α-synuclein could play a role in brain inflammatory responses through modulation of arachidonic acid metabolism and downstream PGD2/PGJ2 production.

As far as we know, we established the first model of inflammation in which the endogenous highly reactive product of inflammation PGJ2, induces PD pathology convincingly (Pierre et al., 2009). Microinfusion of PGJ2 into the brainstem and striatum of adult FVB male mice led to a dose-dependent reduction in the number of dopaminergic (TH+) neurons in the *substantia nigra pars compacta*, with little damage to local GABAergic interneurons, and to the appearance of Lewy-like bodies and activated glial cells. PGJ2-treatment resulted in a PD-like phenotype exhibiting gait disturbance and impaired balance. More recently we showed that this PGJ2-induced mouse model that mimics in part chronic inflammation, exhibits slow-onset PD-like pathology (Shivers et al., 2014). In this mouse model, microglia activation was evaluated *in vivo* by PET with [^11^C](R)PK11195 (Banati, 2002) to provide a regional estimation of brain inflammation. We also demonstrated that PACAP27, a peptide that increases intracellular cAMP levels (Moody et al., 2011), reduced dopaminergic neuronal loss and motor deficits induced by PGJ2, without preventing microglia activation. The latter could be problematic in that persistent microglia activation can exert long-term deleterious effects on neurons and behavior. In conclusion, this PGJ2-induced mouse model is optimal for testing diagnostic tools such as PET, which is a powerful technique to quantitatively assess neuroinflammation *in vivo* (Stoessl, 2014), as well as therapies designed to target the integrated signaling across neurons and microglia, to fully benefit patients with PD.

### Amyotrophic lateral sclerosis (ALS)

There is evidence supporting the involvement of J2 prostaglandins in ALS. As such, 15d-PGJ2 was shown to accumulate in spinal motor neurons of patients with amyotrophic lateral sclerosis (ALS) (Kondo et al., 2002; Zhang et al., 2010). Moreover, astrocytes from mice carrying the *SOD1*G93A mutation were shown to be toxic to stem cell-derived human motor neurons but not to interneurons (Di Giorgio et al., 2008). The astrocyte induced neurotoxicity was mediated by up-regulation of PGD2 signaling, and was prevented by MK05524, an antagonist for the PGD2 receptor DP1 (Di Giorgio et al., 2008). In a more recent study, an *in vivo* genetic approach validated the importance of this DP1-mediated mechanism for neuronal degeneration. As such, genetic ablation of DP1 in *SOD1*G93A mice extended their life span, decreased microglial activation, and reduced motor neuron loss (de Boer et al., 2014). These results suggest that blocking DP1 may be a therapeutic strategy in ALS (Di Giorgio et al., 2008; de Boer et al., 2014).

### Stroke

Stroke and silent brain infarcts are high risk factors for dementia and neurodegenerative diseases such as AD (Vermeer et al., 2003) and PD (Becker et al., 2010; Rodriguez-Grande et al., 2013). The cyclooxygenase pathway was considered to be a valuable therapeutic target for stroke, however while COX-2 inhibitors are able to diminish injury in stroke models, they also produce an unbalance in prostanoid synthesis that promotes damaging vascular effects (Iadecola and Gorelick, 2005). For this reason, new therapeutic strategies targeting the factors that mediate the damage downstream from COX-2 may offer stroke patients powerful new tools to ameliorate brain damage and improve their functional outcome (Iadecola and Gorelick, 2005). Some of these factors could be J2 prostaglandins, as their levels in the brain are highly elevated in rodent models of cardiac arrest and stroke (Liu et al., 2011, 2013a,c; Shaik et al., 2014). Due to their inhibitory effects on the UPP, J2 prostaglandins could play an important role in brain ischemia brought about by cardiac arrest or stroke, as UPP impairment is highly relevant to these conditions (Caldeira et al., 2014). Thus, therapeutic strategies targeting the deleterious effects of J2 prostaglandins could offer great promise.

### Traumatic brain injury (TBI)

TBI is another neurological condition associated with J2 prostaglandins. In rodents, TBI elevates J2 prostaglandin levels in the brain to concentrations similar to those shown to be neurotoxic *in vitro* (Kunz et al., 2002; Hickey et al., 2007). TBI initiates an inflammatory cascade that leads to acute pathologic processes as well as long-term neuronal damage (Ziebell and Morganti-Kossmann, 2010). The nuclear receptor PPARγ, for which 15d-PGJ2 is an endogenous ligand, is considered a major anti-inflammatory and neuroprotective target for treating patients with TBI. However, PPARγ activation can also trigger apoptosis (Qi et al., 2010). These opposing effects seem to be related to the level of PPARγ agonists produced (Clay et al., 1999; Na and Surh, 2003). Low PPARγ agonist levels exert neuroprotective and anti-inflammatory effects that include down-regulation of inflammatory responses, reduction of oxidative stress, inhibition of apoptosis, and promotion of neurogenesis, while high levels induced apoptosis (Qi et al., 2010). The regulatory mechanisms and signaling cascades underlying the opposing PPARγ effects require further elucidation (Qi et al., 2010).

### Krabbe disease

This disease is associated with demyelination, for which the *twitcher* mouse is an authentic animal model (Duchen et al., 1980; Kobayashi et al., 1980). In this mouse model, myelination proceeds normally up to post-natal day 30, when demyelination initiates due to oligodendrocyte apoptosis accompanied by microglia activation and astroglyosis (Mohri et al., 2006). Remarkably, a blockade of the PGD2 signaling cascade in the *twitcher* mouse via knock-out of the PGD2 synthase H-PGDS or the PGD2 receptor DP1, or treating these mice with the H-PGDS inhibitor HQL-79 [4-benzhydryloxy-1-[3-(1*H*-tetrazol-5-yl)-propyl]piperidine], suppressed astroglyosis, demyelination, twitching and spasticity. These results support the notion that PGD2 and perhaps its metabolites, are key to the pathological demyelination occurring in the *twitcher* mouse, and the neuroprotective potential of manipulating the PGD2-signaling to overcome demyelination in Krabbe disease (Mohri et al., 2006; Bosetti, 2007).

## Potential J2 prostaglandin therapeutic targets

While inflammation can be beneficial, failure to adequately control its abatement when the injurious agent is neutralized, is increasingly believed to be one of the major causes of chronic inflammation (Gilroy, 2010). The therapeutic potential of the prostanoid pathway is supported by the clinical efficacy of NSAIDs, although they present risky side effects as current NSAIDs non-selectively inhibit the overall synthesis of all prostaglandins (Table 3).

**Table 3:** **Potential J2 prostaglandin therapeutic targets**.

**Target**	**Drugs**	**Effects**	**References**
Cycloxygenases (COXs)	NSAIDS	COX inhibitors. Prevent/diminish neuroinflammation. Inhibit synthesis of all PGs. Exhibit cardiovascular, gastrointestinal and other side effects.	Iadecola and Gorelick, 2005; Rainsford, 2007; Ng and Chan, 2010
Monoacyl-glycerol lipase (MAGL)	JZL184	MAGL selective and irreversible inhibitor. Prevents/diminishes neuroinflammation. Inhibits synthesis of all PGs in the brain. No detectable gastrointestinal side effects.	Nomura et al., 2011; Legg, 2011
H-PGDS	HQL-79	H-PGDS inhibitor. Prevents demyelination, astroglyosis and spasticity in the *twitcher* mouse. Potential for treating Krabbe disease.	Mohri et al., 2006; Bosetti, 2007
DP1	Agonists	Effective against stroke.	Ahmad et al., 2010
	Antagonists	Treat ALS, Krabbe disease and pain.	Mohri et al., 2006; Di Giorgio et al., 2008; Jones et al., 2009; de Boer et al., 2014;
DP2	Antagonists	Effective anti-inflammatory drugs (for asthma and allergies). Potential for treating neurodegenerative diseases and pain.	Jones et al., 2009; Norman, 2014
PPARy	Agonists	Effective against stroke, TBI, spinal cord injury, multiple sclerosis, AD, PD.	Combs et al., 2000; Diab et al., 2002; Kapadia et al., 2008; Nolan et al., 2013
Prostaglandin TA Transporter (PGT)	T26A	Highly selective PGT competitive inhibitor. Prolongs prostaglandin half-life.	Chi et al., 2011
Michael addition	PAPCAP27	Increases intracellular cAMP. Protective for stroke, PD, HD and TBI. Prevents PGJ2-induced neurodamage *in vitro* and *in vivo* (PD model).	Reglodi et al., 2004; Metcalfe et al., 2012; Shivers et al., 2014
	Lipocardium	Negatively charged liposomes to deliver PGA2 to activated arterial wall lining cells to reduce atherosclerosis.	Homem de Bittencourt et al., 2007

A more recent approach of diminishing prostaglandin-induced neuroinflammation is to block the activity of the enzyme monoacylglycerol lipase (MAGL) (Table 3). MAGL, which hydrolyzes endocannabinoids, can also regulate arachidonic acid release in the brain but not in the gastrointestinal tract, that culminates in the generation of neuroinflammatory prostaglandins (Nomura et al., 2011; Piro et al., 2012). A novel MAGL selective and irreversible inhibitor (JZL184, which is 4-nitrophenyl-4-[bis(1,3-benzodioxol-5-yl)(hydroxy)methyl]piperidine-1-carboxylate) has shown promising results for blocking the neuroinflammation in the brain associated with PD and other neurodegenerative disorders (Legg, 2011; Nomura et al., 2011).

Additional specific targets in the prostaglandin pathways, such as prostaglandin synthases, prostaglandin transporters, and prostaglandin receptors have also emerged as drug targets (Table 3). For example, recently a highly selective competitive inhibitor of the prostaglandin transporter PGT was found to prolong PGE2 half-life, *in vitro* and *in vivo* (Chi et al., 2011). It is expected that further delineation of the prostaglandin pathway will yield novel beneficial therapeutics in the years to come (Smyth et al., 2009).

As discussed throughout this review, further knowledge on the neurotoxic mechanisms mediated by J2 prostaglandins and their contribution to the progression and longevity/resolution of the inflammatory response are needed to develop novel and more effective neuroprotective therapeutic strategies to attenuate inflammation. J2 prostaglandins represent attractive therapeutic targets because of their important roles in the development and resolution of inflammation. In particular, J2 prostaglandin-dependent therapeutics should target mechanisms of action that include receptor activation and Michael addition.

### Receptor modulation (Table 3)

It is critical to fully understand the dual roles of these prostaglandins as pro- and anti-inflammatory agents. The opposing effects of PGD2/J2 prostaglandins on inflammation are reflected by the responses induced by activation of the DP1 or DP2 receptors, for which a range of antagonists are undergoing clinical evaluation (Pettipher et al., 2007; Sandig et al., 2007; Ricciotti and FitzGerald, 2011). Some of these potential drugs can reach the cerebrospinal fluid upon oral administration (Pettipher et al., 2007). While DP1 agonists seem to alleviate brain damage upon stroke (Ahmad et al., 2010), DP1 antagonists are being proposed to treat disorders such as ALS (de Boer et al., 2014) and Krabbe disease (Mohri et al., 2006). DP2 antagonists are highly relevant to treating allergies and asthma (Norman, 2014). The effective anti-inflammatory properties of these drugs may be relevant to blocking inflammation associated with neurodegeneration and pain (Jones et al., 2009).

PPARγ agonists seem to protect neurons not only following acute CNS injury including stroke, spinal cord injury and TBI after which massive inflammation plays a detrimental role, but also in neurodegenerative conditions including multiple sclerosis (Diab et al., 2002; Chaudhuri, 2013), AD (Combs et al., 2000) and PD (Kapadia et al., 2008). While 15d-PGJ2 is thought to be the endogenous ligand for PPARγ, the thiazolidinediones (TZDs) are used as potent exogenous agonists exerting their neuroprotective effects via prevention of microglial activation, inflammatory cytokine and chemokine expression, and promoting the anti-oxidant mechanisms in the injured CNS (Kapadia et al., 2008). More recent evidence suggests that the nuclear receptor (NR) superfamily of transcription factors including Nuclear receptor-related factor1 (Nurr1), PPARs, retinoic acid and glucocorticoid receptors show promise as therapeutic targets for PD. Since they are known to regulate an array of inflammatory mediators, it is postulated that modulating Nurr1 expression or NR receptor activation, including PPARs, via agonists would protect against dopaminergic neuronal death induced by inflammation (Nolan et al., 2013).

Overall, the effectiveness of J2 prostaglandin receptor agonists/antagonists on the treatment of neurodegenerative conditions needs to be carefully investigated as these receptors may act sequentially to initiate and sustain disease states, and/or play complementary roles. Potentially, a combination of these receptor agonists/antagonists could prove to be a very promising therapeutic approach (Jones et al., 2009). Whether these drugs can be applied to preventing chronic long-term neuroinflammation remains to be explored.

### Michael addition (Table 3)

The best characterized mechanism of action of J2 prostaglandins is the covalent modification of proteins at cysteine residues through Michael addition, which is attributed to their electrophilic nature (Oeste and Perez-Sala, 2014). In this regard, proteomic approaches used in the past few years have provided much needed knowledge about the pharmacological actions and signaling mechanisms of J2 prostaglandins (Oeste and Perez-Sala, 2014). Detailed investigation of the protein targets directly affected by these lipid mediators of inflammation, is critical to the development of more specific and effective therapeutic approached against the deleterious effects of neuroinflammation. Identification of these protein targets will provide important clues on the pathways modulated by J2 prostaglandins and the mechanisms underlying their beneficial or deleterious effects.

As we discussed above, two of these pathways that are affected by J2 prostaglandins and that are highly relevant to neurodegeneration are the UPP and mitochondrial function. We recently investigated *in vitro* (Metcalfe et al., 2012) and *in vivo* (Shivers et al., 2014) a potential therapeutic approach to overcome J2 prostaglandin neurotoxicity, based on elevating intracellular cAMP with PACAP27 (pituitary adenylate cyclase-activating polypeptide). PACAP27 is a potent neuroprotective lipophilic peptide in different models of neuronal injury such as stroke, PD, HD, TBI, retinal degeneration, and others, where it exhibits anti-apoptotic, anti-inflammatory and anti-oxidant effects (Reglodi et al., 2004; Atlasz et al., 2010; Ohtaki et al., 2010; Dejda et al., 2011; Mao et al., 2012). While we confirmed the anti-apoptotic effects of PACAP27 in our studies, it was clear that PACAP27 as tested and by itself was not sufficient to overcome all of the neurodamaging effects of PGJ2 (Metcalfe et al., 2012; Shivers et al., 2014). Ideal therapeutic interventions against J2 prostaglandins may require a combinatorial approach to effectively prevent the pleiotropic effects of these highly reactive endogenous mediators of inflammation.

In contrast, negatively charged cyclopentenone prostaglandin-based liposomes (LipoCardium) were developed to specifically deliver prostaglandins, in this case PGA2, to injured arterial wall cells of atherosclerotic mice (Homem de Bittencourt et al., 2007). Anti-inflammatory, anti-proliferative, anti-cholesterogenic and cytoprotective effects were obtained with LipoCardium. This strategy opens up new avenues for specific prostaglandin delivery to humans, including a chronic slow delivery.

In conclusion, much remains to be discovered about the biology of the J2 prostaglandins, to prevent their neurotoxic effects and possibly “trick” ongoing inflammation into resolution. Further investigations may provide important clues “to bring us into a new era of inflammation research, which, if approached with creativity and persistence, might provide numerous benefits for those suffering from inflammation-mediated diseases” (Gilroy, 2010).

### Conflict of interest statement

The authors declare that the research was conducted in the absence of any commercial or financial relationships that could be construed as a potential conflict of interest.
